# Domain Swapping in Allosteric Modulation of DNA Specificity

**DOI:** 10.1371/journal.pbio.1000554

**Published:** 2010-12-07

**Authors:** Chad K. Park, Hemant K. Joshi, Alka Agrawal, M. Imran Ghare, Elizabeth J. Little, Pete W. Dunten, Jurate Bitinaite, Nancy C. Horton

**Affiliations:** 1 Department of Chemistry and Biochemistry, University of Arizona, Tucson, Arizona, United States of America; 2 New England Biolabs Inc., Ipswich, Massachusetts, United States of America; 3 Stanford Synchrotron Radiation Laboratory, Stanford University, Menlo Park, California, United States of America; Brandeis University, United States of America

## Abstract

SgrAI is a type IIF restriction endonuclease that cuts an unusually long recognition sequence and exhibits allosteric self-modulation of cleavage activity and sequence specificity. Previous studies have shown that DNA bound dimers of SgrAI oligomerize into an activated form with higher DNA cleavage rates, although previously determined crystal structures of SgrAI bound to DNA show only the DNA bound dimer. A new crystal structure of the type II restriction endonuclease SgrAI bound to DNA and Ca^2+^ is now presented, which shows the close association of two DNA bound SgrAI dimers. This tetrameric form is unlike those of the homologous enzymes Cfr10I and NgoMIV and is formed by the swapping of the amino-terminal 24 amino acid residues. Two mutations predicted to destabilize the swapped form of SgrAI, P27W and P27G, have been made and shown to eliminate both the oligomerization of the DNA bound SgrAI dimers as well as the allosteric stimulation of DNA cleavage by SgrAI. A mechanism involving domain swapping is proposed to explain the unusual allosteric properties of SgrAI via association of the domain swapped tetramer of SgrAI bound to DNA into higher order oligomers.

## Abstract 2

The structure of two DNA-bound SgrAI enzyme dimers is presented, along with mutagenesis experiments supporting a role for this structure in polymer formation and the activation of DNA cleavage by SgrAI.

## Author Summary

Restriction endonucleases protect their bacterial hosts from viral infection by cleaving any invading viral DNA. One such enzyme, SgrAI, cleaves DNA very slowly but can be activated to cleave DNA 200 times more rapidly. Activation occurs when the enzyme interacts with two or more copies of DNA containing its recognition sequence. We have recently discovered that this enzyme forms polymers when activated. Polymerization may function to sequester the activated enzyme away from, and thereby protect, the host DNA. We have determined the three-dimensional structure of two SgrAI enzymes, each bound to their recognition sequence, interacting in a way that may occur in the polymer. The observed interaction involves a very unusual swapping of parts of each enzyme, termed domain swapping, which has rarely been found in enzyme activation. In support of the idea that this interaction operates during polymer formation and activation of SgrAI, we have shown that mutations designed to interfere with the interaction eliminate both activation and polymerization of the enzyme.

Domain swapping involves the exchange of identical folding motifs between two copies of the same polypeptide chain [1], and as a result, a tight oligomer is formed. Such swapping has been found in many oligomeric proteins, where the swapped form is the biologically natural form [2] and in some cases where binding to a receptor brings two copies of a polypeptide together [3]. Domain swapping can in principle also lead to aggregation, as may occur in some amyloid diseases [4]. Clear cases of reversible domain swapping, serving to alter natural functions such as enzyme activity or specificity, are less well known.

Sequence-specific endonucleases capable of cleaving longer recognition sequences are highly sought for use in genomic work, as longer sequences occur less frequently and allow the manipulation of larger DNA fragments. The type IIF restriction endonuclease SgrAI cleaves a relatively long cognate, primary site sequence, CR|CCGGYG (R = A or G, Y = C or T, | denotes cut site) [5]. However, SgrAI also exhibits unusual biochemical properties; under certain conditions SgrAI cleaves plasmids bearing two copies of its recognition sequence faster than those bearing only a single site [6]–[8]. Further, SgrAI will also cleave at secondary sites containing the sequences CR|CCGGY(A,C,T) and CR|CCGGGG but only appreciably when the plasmid contains a primary site [9],[10]. Secondary sites are distinct from star sites, in that secondary sites are cleaved under solution conditions that are also optimal for cognate sequence cleavage. In contrast, star site sequences are cleaved appreciably only under special reaction conditions, such as high enzyme concentrations or the presence of organic solvents or Mn^2+^, and are discriminated against under optimal enzyme conditions by 2–4 orders of magnitude [11].

Type II restriction endonucleases typically bind and recognize palindromic sequences as dimers [11],[12], but the unusual biochemical properties exhibited by SgrAI suggest the formation of a higher order oligomer, containing altered enzymatic properties. For example, at low enzyme concentrations SgrAI cleaves plasmids bearing one or two sites at equal rates, but higher concentrations of enzyme result in the faster cleavage of the two site plasmid [7],[8]. This suggests that SgrAI forms a tetramer, or higher order species, on the DNA and cleaves two SgrAI sites in a concerted manner, similarly to that reported for the pseudodimer Sau3AI [13]. The event or process that leads to stimulation of DNA cleavage activity must occur through three-dimensional space, as the accelerated and concerted cleavage also occurs with plasmids each bearing a single site but connected by catenation [8]. Cleavage at primary site sequences in plasmids can also be stimulated by the addition of oligonucleotides containing the primary site sequence, intact or mimicking the cleavage products of SgrAI [8]–[10]. Analytical ultracentrifugation (AUC) shows that SgrAI exists as a dimer in the absence of DNA but forms both DNA bound dimers and high molecular mass aggregates in the presence of a 20 base pair DNA containing its recognition site [7]. The stoichiometry of this mixture of species has been determined by titration of DNA with SgrAI in AUC sedimentation velocity experiments showing one dimer of SgrAI per DNA duplex [7]. The DNA cleavage turnover number, k_cat_, of SgrAI with its cognate sequence shows a sigmoidal dependence on SgrAI concentration, consistent with the formation of an activated oligomer at the higher enzyme concentrations [7],[8]. The DNA cleavage rate also shows a sigmoidal dependence on DNA concentration, suggesting that DNA binding stimulates the formation of the activated conformation, which is presumably a tetramer or higher molecular weight species [10].

In addition to the stimulation of cleavage at cognate sequences, CR|CCGGYG, cleavage at secondary sites (CR|CCGGY(A,C,T) and CR|CCGGGG) by SgrAI can also be stimulated using the appropriate conditions. The cleavage at secondary sites is 200-fold slower relative to cognate [9], but this difference is reduced to only 10-fold when the secondary sites are adjacent to DNA ends simulating the products from cognate DNA cleavage [9]. The stimulation also involves an interaction in three dimensions, as stimulation of cleavage at secondary sites can be induced on a plasmid catenated to a plasmid containing the cognate sequence [8].

The related enzymes, Cfr10I [14],[15] and NgoMIV^14^, which cleave sequences R|CCGGY and G|CCGGC, respectively, form stable tetramers in both the presence and absence of DNA. Yet the crystal structures of SgrAI bound to cognate DNA CACCGGTG determined previously show only a dimer of SgrAI bound to a single duplex of DNA [16]. In this structure the central CCGG sequence is recognized by SgrAI using the same side chain-DNA contacts found in the structure of NgoMIV bound to DNA. The degenerate base pairs of the sequence, in the second and seventh positions (CRCCGGYG), appear to be recognized by indirect readout, and the outer base pair (CRCCGGYG) is recognized by a single contact from an arginine side chain to the G. However, these structures did not shed light on the mechanism of activation and sequence modulation seen in the SgrAI cleavage studies. We have recently shown that SgrAI forms oligomers of DNA bound dimers with primary site DNA at sufficient concentrations of enzyme and DNA [17]. These high molecular weight species (HMWS) occur under nearly identical conditions as the stimulation of SgrAI mediated primary and secondary site DNA cleavage. We have proposed that the HMWS are the activated form of SgrAI, or are at least a pre-requisite for the stabilization of the activated form. Here we present a new structure of SgrAI bound to DNA showing the close interaction of two DNA bound dimers. This tetrameric form is completely unlike those of Cfr10I or NgoMIV, as the tetrameric interface is at the opposite side of the dimer and is stabilized by swapping of the amino terminal 24 amino acid residues. A mechanism for the modulation of specificity is postulated and tested by analysis of the effects of mutations designed to destabilize the domain swapped tetramer. The mutant enzymes show complete loss of allosteric stimulation, as well as the inability to form HMWS.

## Results

### Overall Structure

**Figure 1:**
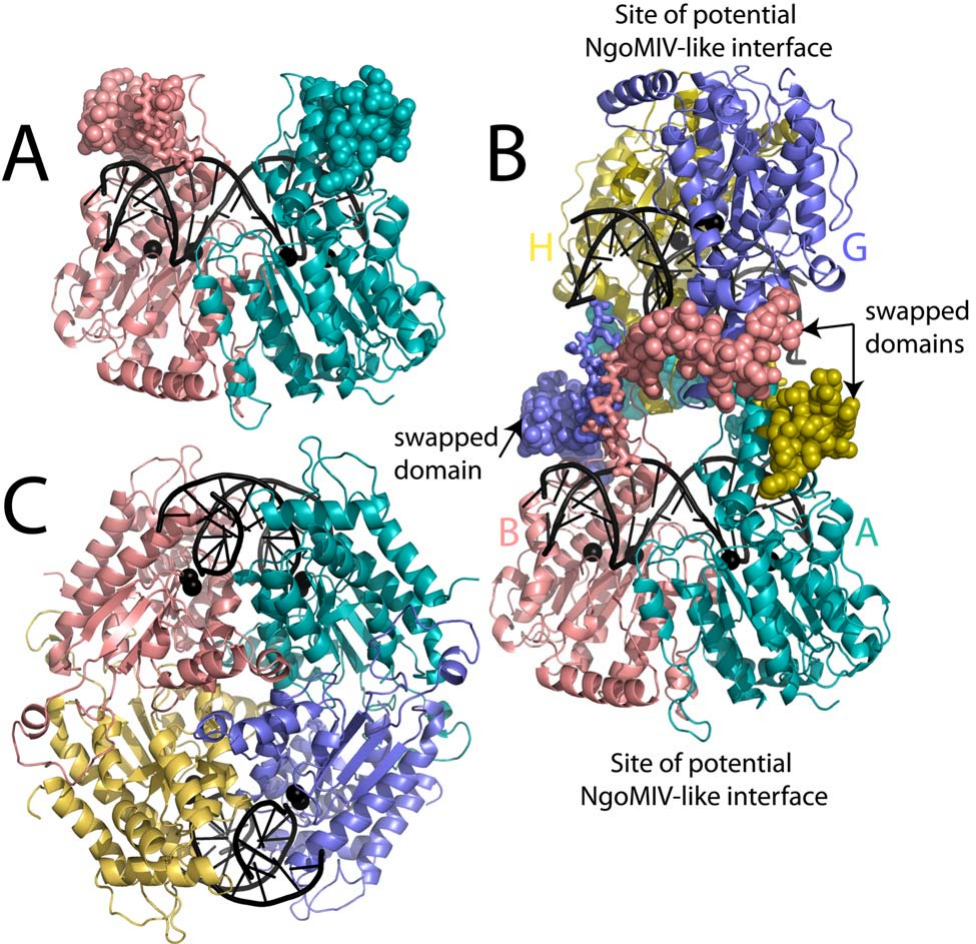
Tetrameric structure of SgrAI. (A) Dimeric structure of SgrAI bound to primary site DNA (PDB code: 3DVO) [16]. Residues of the swapping domain (1–24) shown in space filling spheres. Residues of the hinge loop (25–30) shown as sticks. Residues 31–339 shown as ribbons, and the bound DNA (black) shown as cartoon. Bound Ca^2+^ ions shown as black spheres. (B) Tetrameric structure of SgrAI with subunits A, B, G, and H labeled and colored in teal, salmon, slate, and sand, respectively. Each subunit swaps the amino-terminal 24 amino acid residues (shown as space filling spheres) with those of a subunit in an opposing dimer. Residues of the hinge loop (25–30) shown as sticks. Residues 31–339 shown as ribbons, and the bound DNA (black) shown as cartoon. Bound Ca^2+^ ions shown as black spheres. (C) Ribbon diagram of NgoMIV (PDB code: 1 FIU) (subunits in teal, salmon, slate, and sand) bound to DNA (black) and Mg^2+^ (black spheres).

**Figure 2:**
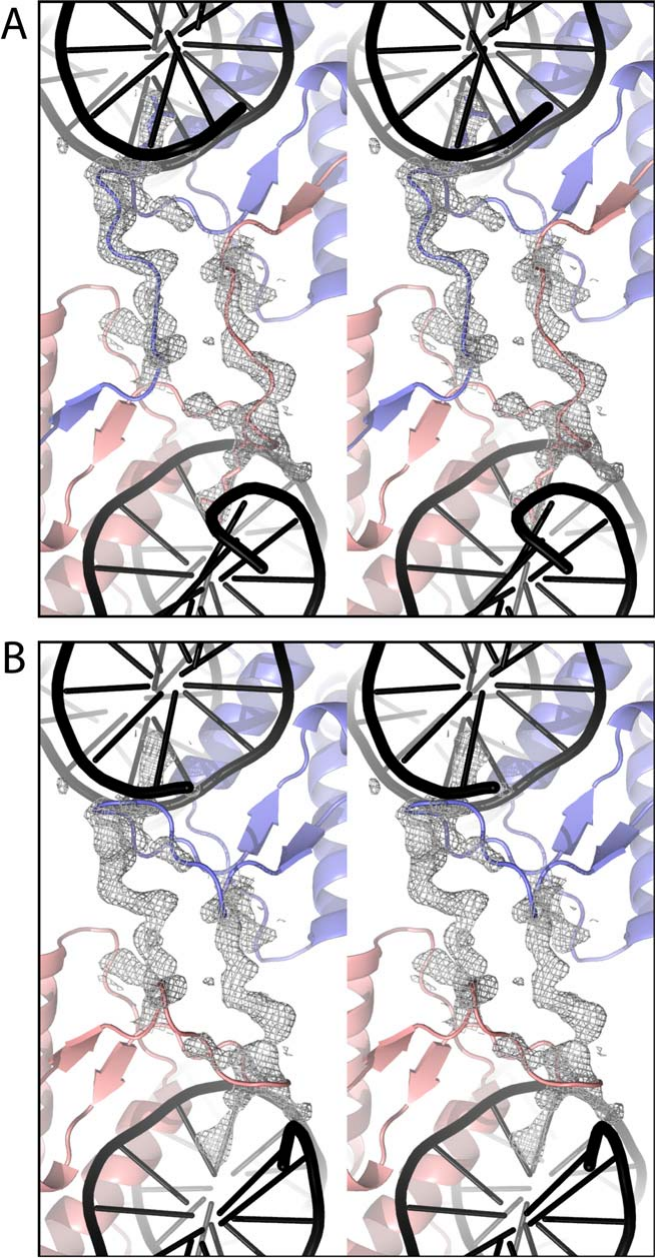
Electron density at swapping hinge loops. (A) 2Fo-Fc SA omit electron density map at 1 σ for residues 23–31 of subunits B (salmon) and G (slate). Ribbon representation of SgrAI subunits shown in swapped conformation. (B) As in (A) with unswapped conformation.

The structure of SgrAI bound to DNA has been refined to 2.03 Å (Table 1) with R_cryst_ of 20.3% and R_free_ of 25.2% and deposited in the RCSB Protein Data Bank with id 3MQ6. The structure shows the same global conformation of the protein as described previously [16], however the amino terminal 24 residues of each subunit appear to be swapped with a subunit of a neighboring dimer (space filling spheres, Figure 1A). The domain swapping, along with other contacts between the two dimers (Figure 1B), create a tetramer of SgrAI bound to two DNA duplexes. The crystallographic asymmetric unit contains two such tetramers. Residues 25–30 of each subunit comprise a linker, or hinge loop as it is referred in domain swapped structures, in the domain swapping, as these residues take on a different conformation in the swapped structures than in the previously described unswapped structures [16]. Figure 2 shows electron density in the vicinity of the swapped domains of subunits B and G, with the trace following a swapped (Figure 2A) or unswapped route (Figure 2B). Electron density for the hinge loop residues of subunits A, D, and E was poor and these residues could not be modeled.

**Table 1:** Diffraction data and structure refinement statistics.

Code	S87
PDB code	3MQ6
Beamline	SSRL BL9-2
Processing program	HKL2000
DNA	17-2 Cognate
Space group	P2_1_2_1_2_1_
Cell	130.38 Å, 134.95 Å, 237.49 Å
Resolution	2.03 Å
Total observations	1,625,899
Unique observations	254,711
% complete	99.6%
I/sigma	30 (2.6)
Multiplicity	3.2 (3.0)
Rmerge^1^	7.1% (69.3%)
Rcryst^2^	20.3%
Rfree^3^	25.2%
Overall B factor (Å^2^, Wilson plot)	37.8
RMSD-bonds	0.023
RMSD-angles	1.07
Asymmetric unit	4 SgrAI dimers/4 DNA duplexes
Numbers of waters	1,296
Number of divalent cations	22 Ca^2+^

1 R_merge_ = Σ_hkl_(|<I_hkl_> − I_hkl_|)/(Σ_hkl_ I_hkl_), where <I_hkl_> is the average intensity over symmetry related and equivalent reflections and I_hkl_ is the observed intensity for reflection hkl.

2 R_cyst_ = Σ_hkl_(||F_obs_| − |F_calc_||)/(Σ_hkl_|F_obs_|) where |F_obs_| and |F_calc_| are the observed and calculated structure factor amplitude for reflection hkl. The sum is carried out over the 98% of the observed reflections which are used in refinement.

3 R_free_ refers to the R factor for the test reflection set (2% of the total observed), which was excluded from refinement.

The domain swapped tetramer of SgrAI is completely unlike the tetramers of NgoMIV or Cfr10I. The SgrAI dimers interact across the DNA binding face using the swapped amino terminal domains (Figure 1A shows the unswapped dimer of SgrAI, Figure 1B shows the domain swapped tetramer of SgrAI). In addition to the swapping interaction, approximately 400 Å^2^ is buried between non-swapping subunits of the tetramer from different dimers (for example, subunits B, salmon, and H, sand). Figure 1C shows a tetramer of NgoMIV bound to DNA, with the subunits of the top dimer (salmon, teal; Figure 1C) oriented as those of the bottom dimer of SgrAI (salmon, teal; Figure 1B), illustrating the different interfaces between dimers in the two tetramers. The contacts to the recognition sequence, CACCGGTG, by SgrAI are identical to those described previously [16]. The crystals were grown in a solution containing 50 mM CaCl_2_, and two Ca^2+^ are bound in each active site at positions found previously [16].

**Figure 3:**
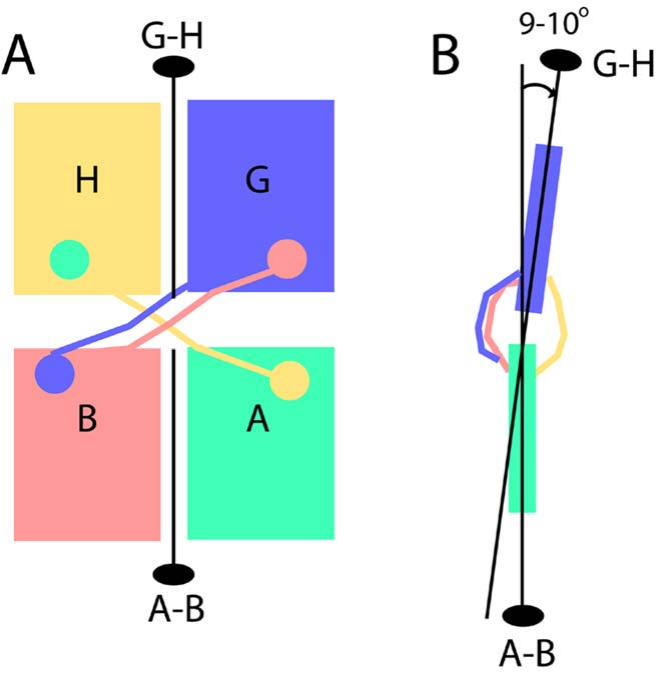
Geometry of SgrAI tetramers. (A) Positions of 2-fold rotational axes of each dimer in tetramer 1, composed of subunits A, B, G, and H, shown as black lines with a black oval. Subunit colors as in Figure 1A–B. Swapped domains and ordered hinge loops represented by circles and lines with the color of their parent subunit. The hinge loop of subunit A is not ordered. (B) Side view of the tetramer shown in Figure 3A showing the positions of the 2-fold axes. The 2-fold dimeric axis of the upper dimer (subunit G, slate; subunit H, sand; Figure 3A) is 9° from that of the lower dimer (subunit A, teal; subunit B, salmon; Figure 3A) and 10° in tetramer 2.

Analysis of the 2-fold axes in the two tetramers of the asymmetric unit reveals a slight difference, corresponding to a 2.5° rotation of one dimer relative to the other. The symmetry within each tetramer shows a small deviation from perfect 222 symmetry, where a total of three 2-fold axes occur orthogonal to one another. The deviation occurs in that the 2-fold axes that relate the two subunits of each dimer in the same tetramer are not coincident and instead are 10° apart (Figure 3A–B). This leads to a slight tilting of one dimer relative to the other in each tetramer (Figure 3B). A difference also exists in the hinge loops (residues 25–30) that connect the swapped domain (residues 1–24) with the rest of each subunit (31–339). In both tetramers, composed of A, B, G, and H in tetramer 1 and E, F, C, and D in tetramer 2, the hinge loops are better ordered in one swapped pair than the other. In tetramer 1 (Figure 3A–B), the hinge loops of swapping pairs B and G are relatively well ordered, while those of A and H are not, and residues 25–30 of subunit A could not be modeled. In tetramer 2, the hinge loops of swapping pairs C and F are well ordered, while those of D and E are not, and residues 25–30 could not be modeled in either subunit. In both tetramers, the better ordered hinge loops occur on the same face of the slightly asymmetric tetramer (Figure 3B). Although the electron density for the hinge loop of subunit A could not be modeled, the electron density for the hinge loop of subunit H allowed modeling in the swapped conformation, suggesting that swapping does occur between subunits A and H. In the absence of defined electron density for the hinge loops of subunits D and E, the possibility exists that these subunits are not domain swapped, yet it should be noted that the hinge loops of the unswapped forms do not show the same degree of disorder [16].

### DNA Binding Assays

**Figure 4:**
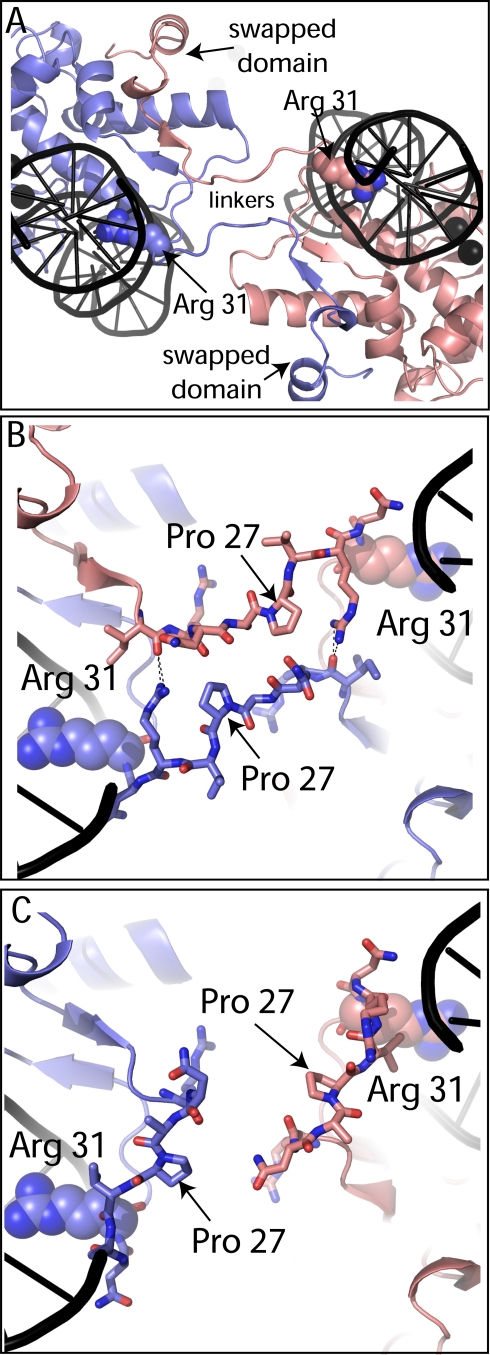
Swapping Hinge loops. (A) Close-up of swapped regions of two subunits in the SgrAI tetramer. Residue Arg 31, responsible for recognition of the outer base pair of the primary site recognition sequence, shown as spheres (subunit B, salmon, blue; subunit G, slate, blue). Active site bound Ca^2+^ ions shown as black spheres and DNA shown in cartoon representation in black. (B) Close-up of interactions at the swapped segments near Pro 27. (C) Same view as in (B) but using unswapped dimer models.

Only the conformations of residues 25–30 differ in the swapped and unswapped conformations of SgrAI (Figure 4); therefore, to test the role of the domain swapped form in the allosteric activity exhibited by SgrAI, two single site substitutions, P27G and P27W, were designed and prepared. First, as a control, the equilibrium dissociation binding constants (K_D_) of purified P27W and of P27G SgrAI were measured using a fluorescence polarization assay (FPA) and fluorophore labeled DNA (Table 2). The assays were performed at 4°C in buffer containing Ca^2+^ ions (20 mM Tris-OAc pH 8.0, 50 mM KOAc, 10 mM Ca(OAc)_2_, 1 mM DTT), which inhibit DNA cleavage while enhancing DNA binding affinity. All data fit well to a 1∶1 binding model without cooperativity. Wild type SgrAI binds to an 18 bp DNA containing a primary site sequence (18-1) with a K_D_ of 0.6±0.2 nM [17]. The mutations P27G and P27W SgrAI weaken the affinity of SgrAI for this DNA, however the affinities are still quite tight (K_D_ = 17±5 nM in the case of P27G, 4.0±0.8 nM in the case of P27W). Binding to PCP (precleaved primary site DNA) appears not to be diminished by any detectable amount by these mutations, with a K_D_ of 6±2 nM for the wild type enzyme [17], compared to 5±2 nM and 9±2 nM for the P27G and P27W SgrAI enzymes, respectively. The binding affinity of the mutant enzymes to secondary site DNA (18-2) was not measured, since native PAGE (see below) indicated very weak binding (K_D_>1 µM).

**Table 2:** Equilibrium dissociation constants (K_D_ (nM)) for wild type SgrAI dimer (unless otherwise noted) and DNA sequences at 4°C.

DNA	WT (nM)	P27W (nM)	P27G(nM)
18-1	0.6±0.2^1^	4.0±0.8	17±5
18-2	2.6±1.2^1^	>1,000	>1,000
PCP	6±2^1^	9±2	5±2

1 From Park et al. (2010) [17].

### Single Turnover DNA Cleavage Assays

Single turnover DNA cleavage rates were measured for P27W and P27G SgrAI with 18 base pair duplex oligonucleotides containing a primary site (18-1) sequence (Table 3). The assays were conducted at 37°C with 1 nM ^32^P labeled 18-1 DNA and 1 µM enzyme, with varying concentrations of added precleaved primary site DNA (PCP). They were also performed side-by-side with those for the wild type enzyme, with careful attention paid to the possible dissociation of PCP into single stranded DNA from repeated freeze thawing. The results show rate constants similar to wild type SgrAI in the absence of PCP (wild type: 0.094±0.015 min^−1^; P27G: 0.06±0.02 min^−1^; P27W: 0.037±0.005 min^−1^). However, while the wild type enzyme is stimulated >200-fold with 1 µM PCP, the mutant enzymes are hardly stimulated at all, 2–3-fold at best. The cleavage rate constants of secondary site DNA by the two mutant enzymes was not measured, as cleavage was undetectable likely due to very weak binding.

**Table 3:** Single turnover DNA cleavage rate constants using 1 µM enzyme.

^32^P Labeled DNA (1 nM)	Conc. Added Unlabeled PCP (nM)	WT SgrAI Rate Constant (min^−1^)	P27W SgrAI Rate Constant (min^−1^)	P27G SgrAI Rate Constant (min^−1^)
1° site (18-1, 18 bp)	0	0.094±0.015^1^	0.037±0.05	0.06±0.02
	10	0.18±0.06^1^	0.028±0.001*	ND
	100	0.30±0.03^1^	0.090±0.001*	ND
	1,000	22±7^1^	0.14±0.01	0.12±0.03

1 From Park et al. (2010) [17].

*Only two repetitions.

### Native Gel PAGE Analysis of HMWS Formation

**Figure 5:**
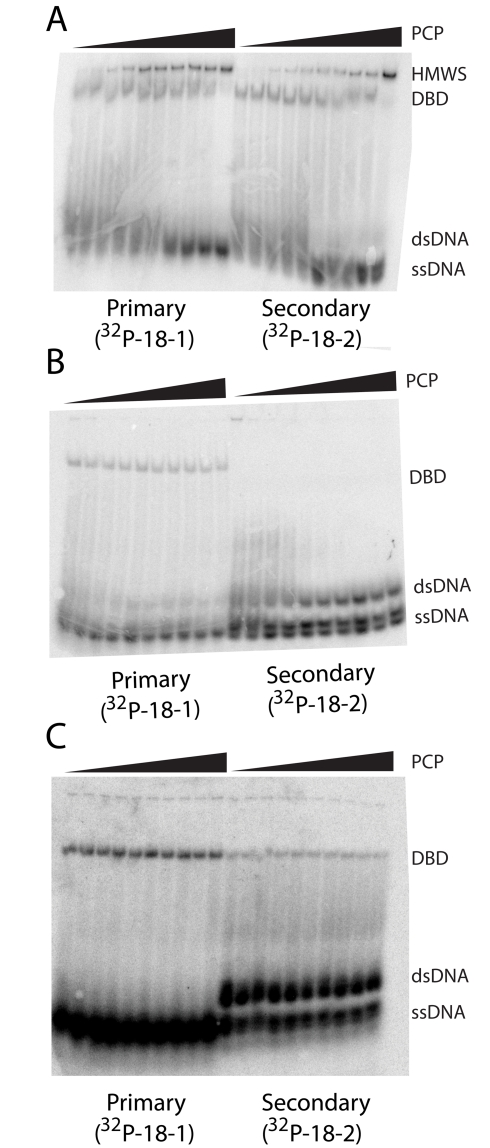
Stimulation of HMWS formation by PCP. Native PAGE of 1 µM wild type or mutant SgrAI with 1 nM ^32^P labeled primary or secondary site (18-1 or 18-2), and increasing concentrations of unlabeled precleaved primary site (PCP, 10, 30, 60, 100, 200, 300, 400, 500, 600, 1,000 nM). (A) wild type SgrAI, (B) P27W SgrAI, (C) P27G SgrAI.

We have used native PAGE to separate different forms of enzyme bound DNA from free DNA and have found two different sizes of enzyme/DNA complexes [17]. The assay utilizes Ca^2+^ in the place of Mg^2+^ to facilitate tight DNA binding without cleavage, just as in the DNA binding affinity measurements, and is also performed at 4°C. We identify the faster migrating species as DNA bound dimer (DBD), and the slower as HMWS, composed of oligomers of DBD [17]. A titration using unlabeled PCP with 1 nM ^32^P labeled 18-1 or 18-2 and 1 µM wild type SgrAI indicates that HMWS forms appreciably at and above 100 nM PCP, and all DBD is shifted to HMWS at 1,000 nM PCP (Figure 5A). However, no HMWS are detected with either mutant enzyme, P27G or P27W SgrAI, under the same conditions (Figure 5B–C). In addition, the mutants appear to bind only weakly to 18-2. The lack of HMWS formation cannot be due to weak PCP binding, as both mutants bind PCP as tightly as wild type (Table 2). With P27W SgrAI, a slowly migrating band is found in lanes with low PCP concentration (Figure 5B) that disappears with higher PCP, hence showing behavior opposite to the HMWS formation by wild type SgrAI. This band may be a result of aggregation of additional SgrAI dimers on the 1∶1 SgrAI/DNA complex, as it disappears with additional PCP where more of the enzyme is expected to be bound to DNA.

## Discussion

Several structures of SgrAI bound to cognate (CACCGGTG) and noncognate (GACCGGTG) DNA, with Ca^2+^ or Mn^2+^, have been determined (Dunten et al. 2008 [16] and current work). In all, SgrAI forms a dimer very similar to those of Cfr10I and NgoMIV. Alignments of the structures show that SgrAI is more similar to Cfr10I, having some small deletions, and several insertions relative to Cfr10I [16]. NgoMIV and Cfr10I form tetramers in the crystal structures, with the tetrameric interface on the side of the dimer opposite to that of the DNA binding site (i.e. tail-to-tail). The new structure of SgrAI described here shows a tetramer that is unlike the NgoMIV and Cfr10I structures, with the tetrameric interface of SgrAI at the DNA binding face of the dimer (i.e. head-to-head) stabilized by the swapping of the amino-terminal 24 residues of each subunit (space filling spheres, Figure 1A). Residues 25–30 comprise the hinge loop that adopts a different conformation in the swapped form (Figures 2, 4). The SgrAI “swapping” domain is absent in NgoMIV and Cfr10I.

The biochemical data suggest that SgrAI can exist in at least two conformations, with one possessing an inherently greater DNA cleavage activity than the other. The observed stimulation of DNA cleavage activity could be accomplished by shifting the equilibrium from the low to the high activity form, possibly stabilized by higher order oligomers that favor the high activity conformation. The rate of DNA cleavage could be controlled by the positioning of groups in the active site, where the optimal alignment results in faster DNA cleavage kinetics. Analysis of the active sites of all SgrAI structures solved to date (Dunten et al. 2008 [16] and current work) shows very similar placement of all groups including the DNA in the various crystal structures, indicating that only a single conformation of the enzyme has been determined, which we have argued to be the low activity conformation [16].

To test the relevance of the domain swapped tetramer in the biochemical activity of SgrAI, two mutants were designed, P27G and P27W, predicted to destabilize the swapped conformation (Figure 4B), through introducing either increased flexibility with the glycine residue or steric conflicts with the large bulky tryptophan side chain. We found that both mutations disrupted the allosteric stimulation of DNA cleavage by SgrAI, without affecting the unstimulated DNA cleavage rate on the primary site sequence (Table 2), and without appreciably affecting binding affinity to uncleaved or precleaved primary site (Table 3). In addition, the activity of P27W SgrAI on plasmids containing either one or two primary site sequences shows that the presence of a second primary site does not appreciably accelerate DNA cleavage, as it does for the wild type enzyme (Text S1, Figures S1–S2). Further, the cleavage pattern of P27W SgrAI does not involve concerted cleavage of the two primary site sequences (Figure S2). Thus the plasmid assays also indicate that P27W SgrAI does not form the activated oligomer proposed to explain the fast, concerted cleavage by wild type SgrAI [6]–[8].

In addition to diminishing the ability of the SgrAI enzyme to be activated in DNA cleavage, the mutations were also found to eliminate the formation of HMWS under conditions where HMWS are formed by wild type enzyme (Figure 5). These results support our previous hypothesis that the HMWS is the activated form of SgrAI [17]. They also support a role for the interface between DBD seen in the structure of the domain swapped tetramer presented here in forming HMWS. Although species as small as tetramers are suggested by the accelerated cleavage of plasmids containing two primary site DNA sequences [6]–[8], our measurement of HMWS formed by wild type SgrAI and primary site containing DNA indicates species much larger than tetramers are formed [17]. Therefore if the tetramer found in the crystal structure is a building block of the HMWS, a second interface between the DNA bound dimers in addition to the domain swapped interface must exist, in order to form run-on oligomers of the size and heterogeneity seen in HMWS; an attractive possibility is the interface used by NgoMIV and Cfr10I (Figure 1B–C).

The effect of the mutations on binding to secondary site DNA was unexpected. Wild type SgrAI binds to both primary and secondary site DNA very tightly, with slightly tighter affinity (∼4-fold) for the primary sequence [17]. Therefore wild type SgrAI seems to discriminate very little between the two sequences at the binding level. Yet these single site substitutions, P27W and P27G, affect affinity very strongly for the secondary, but not the primary, sequence where the K_D_ is shifted from nanomolar to micromolar. The origin of this effect is unknown and requires further investigation.

The SgrAI biochemical and structural data have some similarities to those of another type IIF restriction endonuclease, SfiI [18],[19]. SfiI is a tetramer in solution [19] that cleaves two copies of its recognition sequence in a concerted manner. The crystal structure of SfiI with its recognition sequence DNA show a tetrameric arrangement similar to that of NgoMIV, although the subunit structure is more like the dimeric BglI [18]. The conformation is identified to be in an inactive state since the DNA is mispositioned in the active site and only one of the predicted two divalent cations (Ca^2+^ or Mg^2+^ in the crystal structure) is bound. The low pH of the crystallization conditions may be responsible for the lack of the second divalent cation binding [18]. However, DNA cleavage data show that three recognition sites on the same DNA molecule are cleaved before enzyme dissociation, rather than the predicted two [20]. These data were interpreted as dissociation of one of the two sites cleaved concertedly followed by reassociation and cleavage of the third site prior to enzyme dissociation. Given the model for SgrAI, it is tempting to speculate whether SfiI is fully active also only in oligomers higher order than tetramers, explaining the concerted cleavage of three sites and the inactive conformation of the tetrameric species solved in the crystal structure. However, no direct evidence of oligomerization beyond tetrameric species has been reported for SfiI.

The allosteric communication network has been investigated in Bse634I, another type IIF endonuclease that bears very close structural similarity to SgrAI [21],[22]. Bse634I is a tetramer in solution and cleaves DNA fastest when both DNA binding sites are occupied with its recognition sequence. However, when only a single site is occupied, the DNA cleavage rate is reduced. Hence it possesses both auto-inhibition and stimulation capacities. While we have shown that DNA cleavage by SgrAI is stimulated (>200-fold, 4-fold greater than the 50-fold stimulation of Bse634I), it is not known if auto-inhibition also occurs. For auto-inhibition like that in Bse634I to occur, SgrAI dimers not bound to DNA would need to associate with DNA bound SgrAI dimers and decrease the DNA cleavage rate. Although SgrAI is dimeric in the absence of DNA binding [7], we have shown by gel shift measurements that oligomerization of the SgrAI dimers occurs significantly only with significant concentration of DNA bound dimers (i.e. above 100 nM) and not with excess SgrAI that is not bound to DNA [17]. However, the measurements of the stoichiometry of DNA binding by SgrAI performed with fluorescence anisotropy are suggestive of a second SgrAI dimer binding to the DNA bound SgrAI dimer. The single turnover DNA cleavage assays reported for SgrAI [17] have been done with a substantial excess of SgrAI over the DNA, and if a second SgrAI dimer (without bound DNA) binds to the DNA bound SgrAI dimer, then all reported rate constants have been performed with this additional dimer associated with the enzyme-DNA complex, and with any concomitant auto-inhibition. Investigation of auto-inhibition awaits measurements done with 1∶1 ratios of SgrAI and DNA.

To our knowledge, this is the first clear example of reversible domain swapping functioning to modulate the natural biological activity and specificity of an enzyme. Among previously reported examples [2], that of the RNase enzymes is strongest. RNase A, from bovine pancreas, forms oligomers during lyophilization in acetic acid [23],[24], and the dimers and trimers have been shown to be domain swapped [25]–[27]. Although the conditions for forming the oligomers are artificial, dimerization has been observed at pH 6.5 and 37°C with an equilibrium dissociation constant of 2 mM [28]. The enzymatic activities of the oligomers indicate that hydrolysis of double stranded RNA is faster with the oligomeric forms than with the monomeric, however virtually no difference is seen in the activities of the dimeric and monomeric species [29]. The related bovine seminal RNase, BS-RNase, exists as two interconverting dimers with only one stabilized by domain swapping. The enzyme exhibits cooperativity, but only at very high substrate concentrations (0.3 mM) and the effects are relatively small (1.2–1.3-fold) [30]. The domain swapped form is required for immunosuppression activity, but this activity is not a natural biological function of the enzyme [31]. Therefore, the potential use of domain swapping by SgrAI in a natural function of DNA cleavage rate stimulation and DNA sequence modulation may be the first clear case of a reversible domain swapping used to alter biological activity. This would also be the first case where DNA stimulates such domain swapping.

The unusual DNA cleavage activity of SgrAI may be a consequence of the large genome of *Streptomyces griseus*, from which it is derived. Restriction endonucleases are always coexpressed with a methyltransferase enzyme having the same sequence specificity, which functions to protect the host genome from the cleavage activity of the endonuclease. Hence the SgrAI methyltransferase must methylate all SgrAI recognition sequences within the genome before cleavage by the endonuclease can occur, and this requirement may be difficult due to the large size of the genome (over 8 million bp). The relatively long sequence recognized by SgrAI, 8 bp versus the usual 4–6, may have evolved due to this pressure, since the longer sequence greatly reduces the number of sites to be methylated in the host DNA. In addition, the inherently low cleavage activity of SgrAI in the absence of significant concentrations of unmethylated primary site DNA also reduces the pressure on host DNA, as well as the methyltransferase enzyme. However, such a long recognition sequence will also occur far less frequently in the phage DNA and hence place selective pressure on the enzyme for increased activity in order for adequate protection of the host from phage infection. It appears that one way in which the SgrAI enzyme activity is increased is through the stimulation of its cleavage activity with sufficient concentrations of unmethylated primary site DNA. Another way is through its secondary site cleavage activity, which will induce more cleavages in the phage DNA than at the primary sites alone, and hence could better protect the host. However, to protect against cleavage of the secondary sites in the host genome, the oligomerization may function to sequester activated SgrAI enzymes on the phage DNA and away from the host genome. It may also have an important role in sequestering the phage DNA itself or in rapidly communicating positive allosteric signals to multiple binding sites.

## Methods

### Mutagenesis

A modified USER-friendly DNA engineering method [32] was used to introduce P27W/G codon change into the *sgrAIR* gene. The original USER-friendly DNA mutagenesis technique employs two tail-to-tail overlapping primers, which prime template in the proximity of targeted mutation so that desired nucleotide changes can be incorporated into the primer sequences. The overlapping primers contain a single deoxyuracil (dU) residue flanking the overlap sequence on the 3′ side. After amplification, the dU is excised with the USER enzyme resulting in the PCR product flanked by complementary 3′ single-stranded extensions, which can reanneal to form a recombinant molecule^26^. Archaeal proofreading DNA polymerases are inhibited by dU in the primers; therefore the USER technique is compatible only with *Pfu*Turbo C_x_ Hotstart DNA polymerase (Stratagene), which possesses a genetically modified uracil-binding pocket to overcome inhibition by dU [33]. (*Taq* DNA polymerase is not inhibited by dU, however it is not a proofreading polymerase.) Based on the structural organization of the uracil-binding pocket [33], we rationalized that 5-hydroxymethyluracil (5 hmU) would be prevented from entering the pocket due to the steric clashes with the 5-OH group. Therefore, 5 hmU could, in principle, be used in the primers for DNA amplification with archaeal proofreading DNA polymerases and afterwards be excised from PCR product by human SMUG1 DNA glycosylase, which is specific for 5 hmU [34].

Two overlapping primers, 5′*ATGCGTGGGX*GCGAAATCGTTCCAC and 5′*ACCCACGCAX*TTCGAATATCTTGGATGC, were used to introduce P27W (CCA→TGG) codon change into the *sgrAIR* gene. Likewise, the overlapping primers 5′*ATGCGGGAGX*GCGAAATCGTTCCAC and 5′*ACTCCCGCAX*TTCGAATATCTTGGATGC were used to introduce P27G (CCA→GGA) codon change. Each primer codes for the targeted codon change (underlined) and contains a single 5 hmU residue (marked as “X”) flanking the overlap sequence on the 3′ side (the overlap is shown in *italic*). The entire pET21a_SgrA1R plasmid was amplified as a 7548 bp linear fragment using Phusion DNA polymerase and the corresponding pair of overlapping primers. A 50 µl PCR reaction contained 10 ng of pET21a_SgrA1R template DNA, 0.2 mM dNTPs, 0.2 µM each primer, 3% DMSO, and 0.5 µl of Phusion Hotstart High-fidelity DNA polymerase (New England Biolabs). The pET21a_SgrAI was amplified for 30 cycles using cycling protocol as follows: initial denaturation is 30 s at 98°C; denaturation for 10 s at 98°C, annealing for 20 s at 65°C, polymerization for 4 min at 72°C; and final polymerization is 5 min at 72°C. After completion of the amplification reaction, a 5 µl PCR product aliquot was directly supplemented with 1 µl of 10X NEBuffer 1, 1 µl (20 units) of DpnI restriction endonuclease, and the reaction volume was adjusted to 10 µl with H_2_O. Restriction digestion was carried out for 1 h at 37°C and then reaction was incubated for 20 min at 80°C to inactivate DpnI. Ten units (1 µl) of EndoVIII DNA glycosylase and 10 units (2 µl) of SMUG1 DNA glycosylase (both enzymes from New England Biolabs) were added to the reaction and incubated for 15 min at 37°C to excise 5 hmU residues from PCR product, and then incubated an additional 15 min at room temperature to allow annealing of complementary extensions. *Escherichia coli* T7 Express *I^q^* competent cells (New England Biolabs) were transformed with 5 µl of the annealing reaction. Recombinants were selected by plating 50 µl of transformation reaction on LB plates containing 0.1 mg/ml ampicilin. To confirm nucleotide sequence, plasmid DNA was purified from four individual recombinant colonies and sequenced across the *sgrAIR* gene. No sequence changes, except for the anticipated codon change, were observed.

### Protein Purification

Wild type and mutant SgrAI enzymes were prepared as described [16]. Briefly, the enzymes were expressed in *E. coli* strain ER2566 in the presence of the MspI methyltransferase (New England Biolabs). The enzymes were purified using FPLC (Pharmacia) chromatography and the following chromatographic resins: Heparin FF Sepharose (Pharmacia), SP FF Sepharose (Pharmacia), Q FF Sepharose (Pharmacia), and then a second Heparin FF Sepharose (Pharmacia) chromatographic step. Enzymes were dialyzed into storage buffer (20 mM Tris-OAc, pH 8.0, 50 mM KOAc, 0.1 mM EDTA, 1 mM DTT, 50% glycerol), aliquoted into small single use quantities, flash frozen in liquid nitrogen, and stored at −80°C until used.

### DNA Preparation

**Figure 6:**
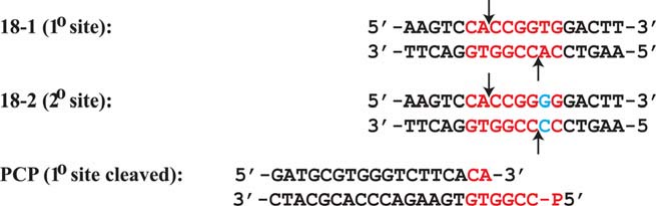
Sequences of DNA constructs. Red, SgrAI primary site recognition sequence; blue, deviation from primary site recognition sequence; arrows, sites of cleavage by SgrAI.

The oligonucleotides (Figure 6) were made synthetically and purified using C18 reverse phase HPLC [35]. The concentration was measured spectrophotometrically, with an extinction coefficient calculated from standard values for the nucleotides [36], and fluorophore where appropriate. Fluorophore labeled DNA included with FLO (6-(3′,6′-dipivaloylfluoresceinyl-6-carboxamido)-hexyl group attached to the 5′ phosphate of the top strand only of PCP) or HEX (6-(4,7,2′,4′,5′,7′-hexachloro-(3′,6′-dipivaloylfluoresceinyl)-6-carboxamido)-hexyl group attached to the 5′ phosphate of both strands of 18-1) were obtained from a commercial synthetic source (Sigma Genosys) and contain a six carbon spacer between the fluorophore and the 5′ phosphate. The self-complementary DNA, or equimolar quantities of complementary DNA, were annealed by heating to 90°C for 10 min at a concentration of 1 mM, followed by slow-cooling to 4°C over 4–5 h in a thermocycler.

DNA used in the crystals have the self-complementary sequence 5′-AAGTCCACCGGTGGACT-3′, identical to 18-1 but one nucleotide shorter on the 3′ side leaving a 5′A overhang. Because freeze-thawing altered the concentration of double stranded DNA used in the assays, DNA used for stimulation of HMWS formation or in single turnover assays was treated very carefully to minimize this problem. Such DNA samples were either reannealed immediately prior to the assay or carefully annealed, assessed for concentration, aliquoted into small amounts, flash frozen in liquid nitrogen, stored at −20°C in water, and used only once after removing from the freezer. DNA was 5′ end labeled with ^32^P using T4 polynucleotide kinase (New England Biolabs) and [γ-32P]-ATP (Perlin-Elmer, Inc.), and excess ATP removed using G-30 spin columns (Biorad Laboratories, Inc.).

### Crystallization, Data Collection, Structure Solution, Refinement, and Analysis

Crystals were prepared with SgrAI and DNA using 1.5 to 3.0 µl of the protein:DNA mixture with 1.0 to 1.5 µl of the precipitating solution (14% PEG 4K, 0.1 M Imidazole (pH 6.5), 0.15 M NaCl, 0.01 M NaNO_3_, 0.05 M CaCl_2_) per drop and placed over 1 ml of the precipitating solution. The SgrAI concentration varied between 10 and 30 mg/ml and was mixed with DNA to give a 1∶2 molar ratio of SgrAI dimer:DNA duplex. Crystals grow overnight to 1 wk at 17°C. The crystals were then exchanged into a cryoprotection solution (25% PEG 4K, 0.1 M Imidazole (pH 6.5), 0.3 M NaCl, and 30% glycerol) and flash-frozen in liquid nitrogen. X-ray diffraction was measured using synchrotron radiation at the Stanford Synchrotron Light Source (SSRL) BL9-2. Data collection was performed while maintaining the crystal at 100K. Image processing and data reduction were performed with HKL2000 (HKL Research, Inc.). The structure was solved by molecular replacement using PHASER [37],[38] and refined using CNS [39], PHENIX [40], REFMAC [41], and the model building program XtalView [42]. Symmetry relations between subunits were determined using LSQKAB [43] as found in CCP4 [44], and the alpha carbon atoms of residues 31–339 of each subunit. The 2Fo-Fc SA omit electron density map was calculated by first deletion of residues 23–31 from each subunit, then performing simulated annealing with a starting temperature of 2,000K in PHENIX [40]. All structure figures were prepared using PYMOL [45].

### Binding Assays

The equilibrium dissociation constant K_D_ of SgrAI-DNA complexes was measured using a fluorescence polarization anisotropy technique [46]. DNA oligonucleotides (1 nM in 2 mL binding buffer: 20 mM Tris-OAc pH 8.0, 50 mM KOAc, 10 mM Ca(OAc)_2_, 1 mM DTT, 10% glycerol) containing a fluorophore (HEX or FLO) ligated to the 5′ end were titrated with increasing amounts of SgrAI enzyme (1 nM–1 µM), and the polarization recorded. Excitation occurred at 537 nm (HEX) or 495 nm (FLO) in a PC1 (ISS instrument) fluorimeter with T format, automatic polarizers, and temperature control. The emitted intensities were measured using a 50.8 mm diameter 570 nm cut-on filter with 580–2,750 nm transmittance range (ThermoOriel Inc., no. 59510) and 1 mm slit widths. The polarization of the emitted light as a function of added enzyme was fit to 1∶1 binding using Kaleidagraph software (Synergy Software) and the following [46]:
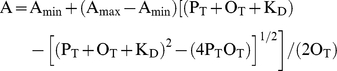
where A is the polarization at a given protein concentration, A_max_ is the predicted polarization of fully bound DNA, A_min_ is the polarization with no protein binding, P_T_ is the total concentration of protein, O_T_ is the total concentration of the DNA, and K_D_ is the dissociation constant to be determined.

### Single Turnover DNA Cleavage Assays

Single turnover measurements of DNA cleavage were performed using chemical rapid quench techniques and 5′-end ^32^P labeled oligonucleotide substrates (typically 1 nM), under conditions of enzyme excess (1 µM), with and without the addition of unlabeled DNA. All reactions were performed at 37°C. For sampling by hand, 5 µl aliquots were withdrawn at specific time intervals after mixing the enzyme and labeled DNA (50 µl each), quenched by addition to 5 ul of quench (80% formamide, 50 mM EDTA), and electrophoresed on 20% denaturing polyacrylamide (19∶1 acrylamide:bisacrylamide, 4 M urea, 89 mM Tris, 89 mM boric acid, 2 mM EDTA) gels. Autoradiography of gels was performed without drying using a phosphor image plate, and exposing at 4°C for 12–17 h. Densitometry of phosphor image plate was performed with a Typhoon Scanner (GE Healthcare Life Sciences) and integration using ImageQuant (GE Healthcare Life Sciences) or ImageJ [47]. The percent of product formed as a function of time was determined by integrating both cleaved and uncleaved DNA bands. The single turnover DNA cleavage rate constant was determined from the data using a single exponential function:
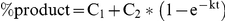
where C_1_ is a constant fitting the baseline, C_2_ is the total percent of DNA predicted to be cleaved by SgrAI, k is the rate constant, and t is the length of incubation in minutes.

### Native Gel Analysis of HMWS Formation

Formation of HMWS was monitored in native gels (8% 29∶1 acrylamide:bisacrylamide in 89 mM Tris base, 89 mM boric acid, and 10 mM Ca^2+^). The electrophoresis running buffer was 89 mM Tris base, 89 mM boric acid, and 10 mM Ca^2+^ and was recirculated during electrophoresis. Gels were electrophoresed in a cold room (4°C) using 190 V. Gels were loaded while undergoing electrophoresis at 400 V, and the voltage returned to 190 V 5 min after the loading of the last sample. Electrophoresis was continued for an additional 2 h. Samples were prepared with 1 µM SgrAI, 1 nM ^32^P labeled DNA, and varied concentrations of unlabeled DNA in binding buffer (20 mM Tris-OAcpH 8.0, 50 mM KOAc, 10 mM Ca(OAc)_2_, 1 mM DTT, 10% glycerol) and incubated for 30 min at 4°C prior to electrophoresis. Autoradiography of gels was performed without drying using a phosphor image plate and exposing at 4°C for 12–17 h. Densitometry of phosphor image plate was performed with a Typhoon Scanner (GE Healthcare Life Sciences) and integration using ImageQuant (GE Healthcare Life Sciences) or ImageJ [47]. Integrated band intensities were normalized using the sum of the DNA bound species (DBD and HMWS) to determine the percent HMWS and then plotted versus PCP concentration using Kaleidagraph (Synergy Software).

## Supporting Information

Figure S1 **Cleavage of single primary site containing plasmid DNA (pMLE2) with wild type or P27W SgrAI.** (A) Image of ethidium bromide stained agarose gel from electrophoresis of reaction products. Lane 1: Molecular weight standard; Lane 2: pMLE2 DNA; Lanes 3–8: 20 nM pMLE2 DNA incubated with 1 µM wild type enzyme at 37°C at 1, 5, 10, 20, 30, 40, 50, and 60 min; Lanes 9–18: 20 nM pMLE2 DNA incubated with 1 µM P27W enzyme at 37°C at 1, 5, 10, 20, 30, 40, 50, and 60 min. Positions of nicked or open circle DNA (OC), linear DNA (L), and supercoiled (SC) marked as indicated. (B) Plot of reaction products as defined in (A) in terms of the percent of the total DNA per lane with wild type SgrAI as a function of length of incubation. (C) Plot of reaction products as defined in (A) in terms of the percent of the total DNA per lane with P27W SgrAI as a function of length of incubation.
(4.48 MB DOC)

Figure S2 **Cleavage of plasmid DNA containing two primary site sequences (pMLE3) with wild type or P27W SgrAI.** (A) Image of ethidium bromide stained agarose gel from electrophoresis of reaction products. Lane 1: Molecular weight standard; Lane 2: pMLE3 DNA; Lanes 3–8: 20 nM pMLE3 DNA incubated with 1 µM wild type enzyme at 37°C at 1, 5, 10, 20, 30, 40, 50, and 60 min; Lanes 9–18: 20 nM pMLE3 DNA incubated with 1 µM P27W enzyme at 37°C at 1, 5, 10, 20, 30, 40, 50, and 60 min. Positions of nicked or open circle DNA (OC), linear DNA (L), supercoiled (SC), and the two products following double cleavage of the plasmid (P1, P2) marked as indicated. (B) Plot of reaction products as defined in (A) in terms of the percent of the total DNA per lane with wild type SgrAI as a function of length of incubation. (C) Plot of reaction products as defined in (A) in terms of the percent of the total DNA per lane with P27W SgrAI as a function of length of incubation.
(4.86 MB DOC)

Text S1 Cleavage of plasmids with one or two primary site sequences by wild type and P27W SgrAI.
(0.04 MB DOC)

## Abbreviations

AUC: analytical ultracentrifugation
DBD: DNA bound dimer
DTT: dithiothreitol
EDTA: ethylenediaminetetraacetic acid
FLO: fluorescein moiety
FPA: fluorescence polarization anisotropy
HEX: hexachlorofluorescein moiety
HMWS: high molecular weight species
OAc: acetate
PAGE: polyacrylamide gel electrophoresis
PC: pre-cleaved primary site DNA without 5′ end at the cleavage site phosphorylated
PCP: pre-cleaved primary site DNA with 5′ end at the cleavage site phosphorylated
