# Supplementary figures and images for: Domain Swapping in Allosteric Modulation of DNA Specificity
Source: PLoS Biol. 2010 Dec 7;8(12):e1000554. doi: 10.1371/journal.pbio.1000554 (PMC2998434; doi:10.1371/journal.pbio.1000554)

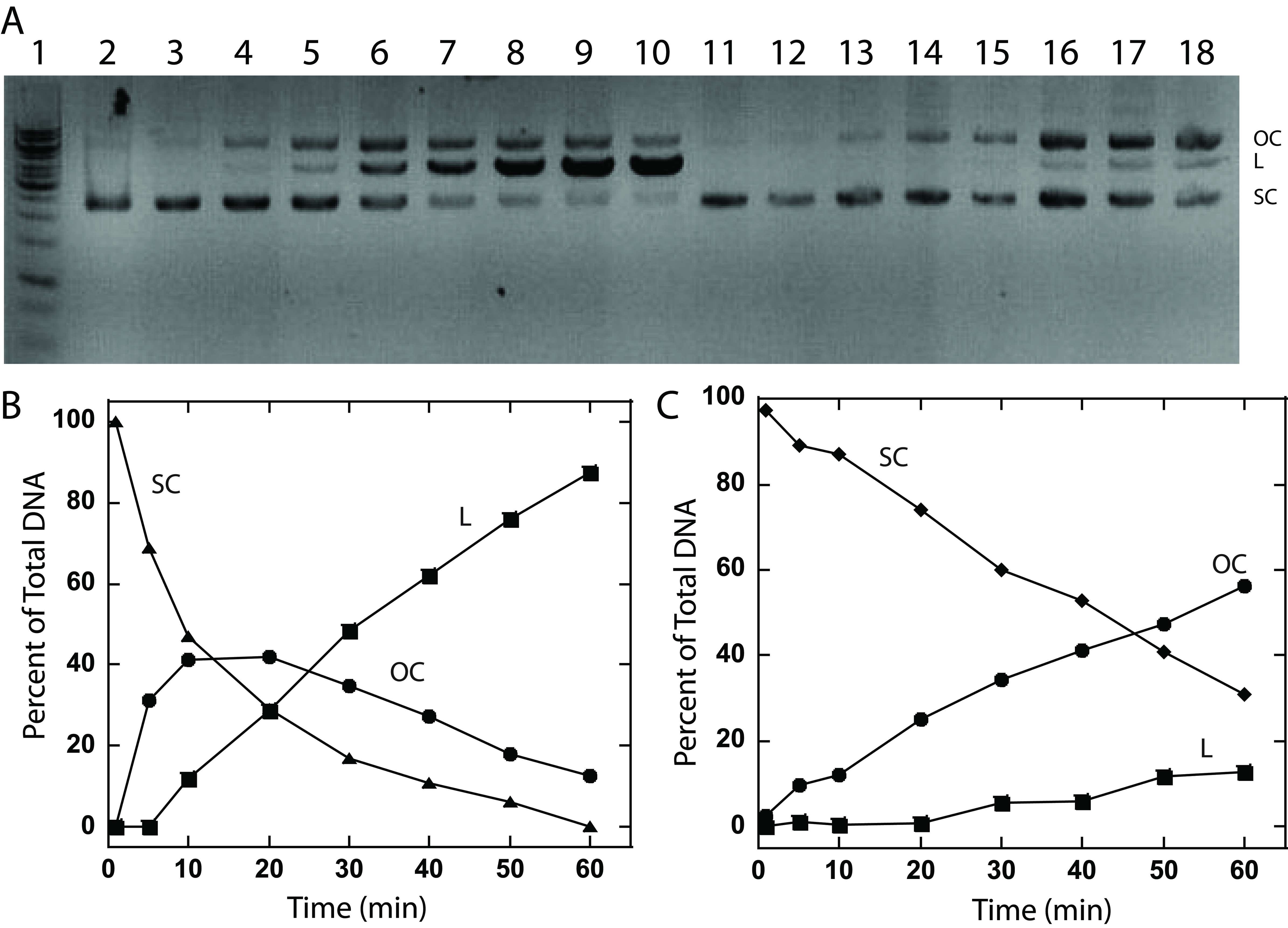

Supplement: Figure S1 — Cleavage of single primary site containing plasmid DNA (pMLE2) with wild type or P27W SgrAI. (A) Image of ethidium bromide stained agarose gel from electrophoresis of reaction products. Lane 1: Molecular weight standard; Lane 2: pMLE2 DNA; Lanes 3–8: 20 nM pMLE2 DNA incubated with 1 µM wild type enzyme at 37°C at 1, 5, 10, 20, 30, 40, 50, and 60 min; Lanes 9–18: 20 nM pMLE2 DNA incubated with 1 µM P27W enzyme at 37°C at 1, 5, 10, 20, 30, 40, 50, and 60 min. Positions of nicked or open circle DNA (OC), linear DNA (L), and supercoiled (SC) marked as indicated. (B) Plot of reaction products as defined in (A) in terms of the percent of the total DNA per lane with wild type SgrAI as a function of length of incubation. (C) Plot of reaction products as defined in (A) in terms of the percent of the total DNA per lane with P27W SgrAI as a function of length of incubation. (4.48 MB DOC) [file pbio.1000554.s001.tif]

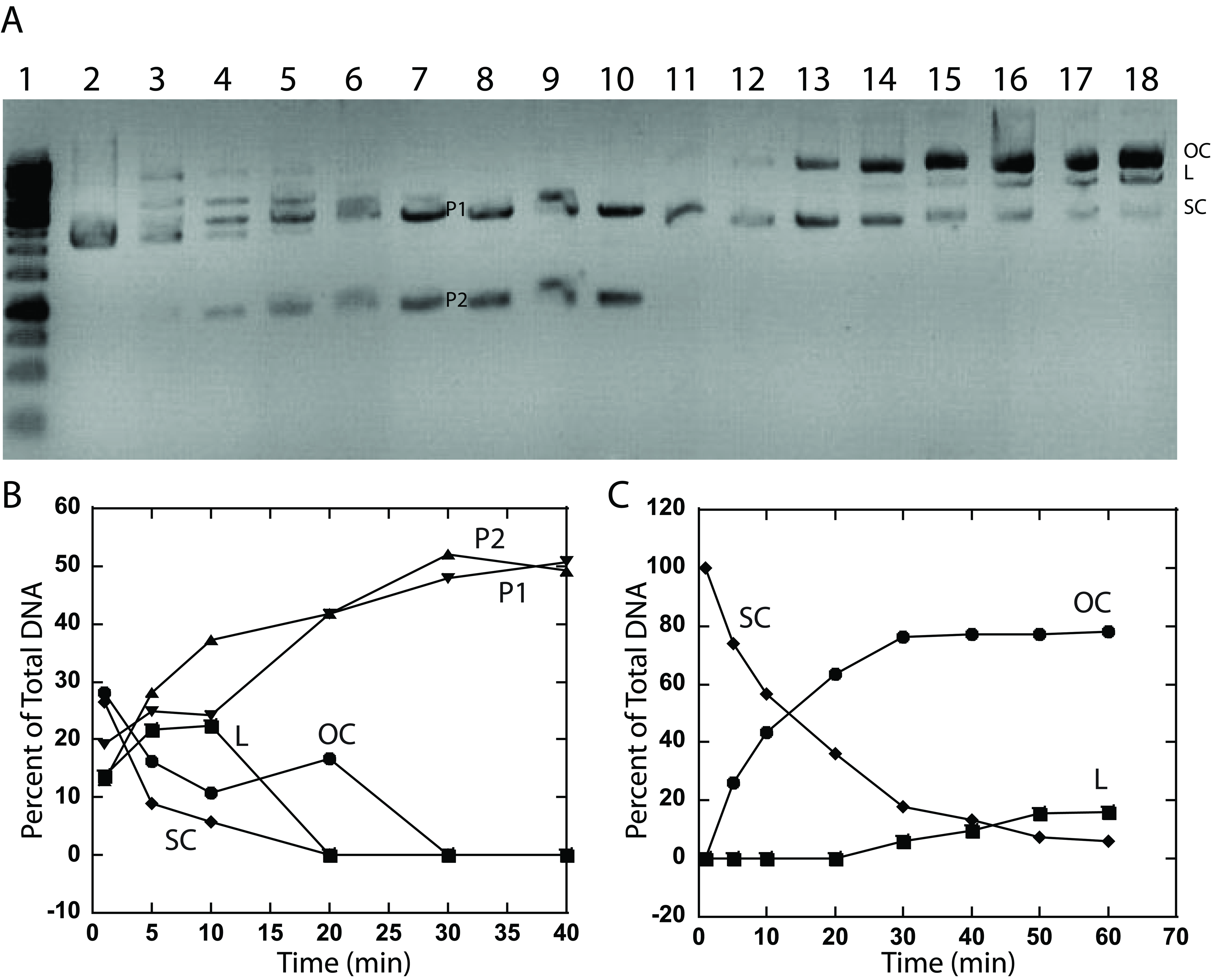

Supplement: Figure S2 — Cleavage of plasmid DNA containing two primary site sequences (pMLE3) with wild type or P27W SgrAI. (A) Image of ethidium bromide stained agarose gel from electrophoresis of reaction products. Lane 1: Molecular weight standard; Lane 2: pMLE3 DNA; Lanes 3–8: 20 nM pMLE3 DNA incubated with 1 µM wild type enzyme at 37°C at 1, 5, 10, 20, 30, 40, 50, and 60 min; Lanes 9–18: 20 nM pMLE3 DNA incubated with 1 µM P27W enzyme at 37°C at 1, 5, 10, 20, 30, 40, 50, and 60 min. Positions of nicked or open circle DNA (OC), linear DNA (L), supercoiled (SC), and the two products following double cleavage of the plasmid (P1, P2) marked as indicated. (B) Plot of reaction products as defined in (A) in terms of the percent of the total DNA per lane with wild type SgrAI as a function of length of incubation. (C) Plot of reaction products as defined in (A) in terms of the percent of the total DNA per lane with P27W SgrAI as a function of length of incubation. (4.86 MB DOC) [file pbio.1000554.s002.tif]
