# Supplementary material for: Domain Swapping in Allosteric Modulation of DNA Specificity
Source: PLoS Biol. 2010 Dec 7;8(12):e1000554. doi: 10.1371/journal.pbio.1000554 (PMC2998434; doi:10.1371/journal.pbio.1000554)
Supplement: Text S1 — Cleavage of plasmids with one or two primary site sequences by wild type and P27W SgrAI. (0.04 MB DOC) [file pbio.1000554.s003.doc]

**Test S1**

**1. Materials and Methods**

Plasmid DNA containing a single primary site (pMLE2) or two primary sites (pMLE3) were prepared from *E. coli* HB101 (obtained from Prof. Stephen Halford) using Qiagen midiprep kits (Qiagen, Inc.) and were stored at -20⁰C until used. Reactions were performed in kinetic buffer (20 mM Tris-Acetate pH 8.0, 50 mM KOAc, 10 mM Mg(OAc)_2_, 1 mM DTT) at 37⁰C with 20 nM plasmid DNA and 1 µM wild type or P27W SgrAI in 100 µl total reaction. Reactions were initiated by the addition of the enzyme, and 10 µl of the reaction was removed and mixed with quench (4 M urea, 50 mM EDTA) at the specified times. The DNA from these quenched reactions was analyzed by electrophoresis on 1% agarose in 1X TAE buffer (40 mM Tris, 20 mM acetic acid, 1 mM EDTA). The DNA was visualized after staining with 0.5 µg/ml ethidium bromide using a UV transilluminator. A digital image of the gel was used to quantitate the amount of DNA in each band using ImageQuant software (Molecular Dynamics, Inc.). Plots were prepared, and rate constants determined, with Kaleidagraph (Synergy Software, Inc.).

**2. Results**

Figure S1 shows the results with pMLE2 DNA, a plasmid which contains only a single primary site. Figure S1A is an image of the agarose gel used to separate the different DNA species. Lane 1 contains a molecular weight marker (1 kb ladder, Fermentas), Lane 2 contains the purified plasmid DNA prior to mixing with enzymes, Lanes 3-10 contain the DNA after incubating with wild type SgrAI for different lengths of time, and Lanes 11-18 contain the DNA after incubating with P27W SgrAI. The positions of the bands corresponding to supercoiled (SC), nicked open circle (OC) and linear DNA (L) are as indicated. Figure S1B shows the plot of the percent of the different DNA species after incubation with wild type SgrAI for the specified amount of time. The amount of supercoiled DNA (SC) decreases rapidly, with nicked open circle (OC) forming early and linear (L) DNA forming later. Using a fit to a single exponential function, the decrease of supercoiled DNA as a function of time gives a rate constant of 0.077 min^-1^. Figure 1C shows the results of the DNA cleavage after incubation with P27W SgrAI. Linear DNA (L, Fig. S1C) is visible after 20 minutes, although more of the DNA is cleaved to the open circle form (OC, Fig. S1C). Fitting to a single exponential function, the decrease of supercoiled DNA as a function of time gives a rate constant of 0.012 min^-1^.

Figure S2 shows the results of DNA cleavage reactions with pMLE3 DNA, a plasmid which contains two primary site sequences. Figure S2A is an image of the ethidium bromide stained gel used to separate and quantitate the different DNA species. Lane 1 is the molecular weight marker (1 kb ladder, Fermentas) and Lane 2 is the pMLE3 DNA prior to incubation with the enzymes. Lanes 3-11 show the results after incubation with the wild type enzyme for 1, 5, 10, 20, 30, 40, 50, and 60 minutes, while Lanes 12-18 are those with P27W SgrAI. Supercoiled (SC), nicked open circle (OC), and linear (L) DNA are marked, as are the two products after dual cleavage of the plasmid, P1 and P2. Figure S2B plots the percent of each DNA species as a function of length of incubation for the reaction with wild type SgrAI, and Figure S2C for P27W SgrAI. Supercoiled (SC) DNA is reduced to 26% after the first minute of incubation with the wild type enzyme (Fig. S2B). Fitting to a single exponential function, the decrease in supercoiled DNA gives a rate constant of 1.4 min^-1^, although the fit is poor suggesting more than one type of reaction contributes to the decrease in supercoiled DNA. Both linear (L, Fig. S2B), resulting from a single double stranded cleavage, and products from dual double stranded cleavage (P1, P2, Fig. S2B) are visible after the first minute of incubation in the case of the wild type enzyme. A small amount of open circle DNA (OC, Fig. S2B) is seen in the reactions with wild type SgrAI, but only early in the timecourse (1-10 minutes). Only linear DNA (L, Fig. S2C) or open circle (OC, Fig. S2C) are formed by the P27W SgrAI in the first 60 minutes of incubation. A fit to a single exponential gives a rate constant for the decrease in supercoiled DNA in the P27W SgrAI reactions of 0.06 min^-1^.

**3. Discussion**

DNA cleavage assays with plasmids containing either a single (pMLE2) or two (pMLE3) primary site sequences were employed to test the effects of the presence of a second primary site on the DNA cleavage rate by P27W SgrAI. The assays were conducted in parallel with the wild type enzyme, which has been reported to exhibit an accelerated DNA cleavage rate on the two-site plasmid relative to the one-site plasmid [1,2]. The results are shown in Figures S1-S2. After one hour incubation, P27W SgrAI produced mostly open circle DNA cleavage products, which result from cleavage of only a single strand of DNA(Lane 18, Fig. S1A, OC, Fig. S2C), however approximately 13% of the DNA is cleaved in both strands at the same site to product linear DNA (L, Lane 18, Fig. S1A, L, Fig. S1C). The same plasmid DNA is converted primarily to linear (L, lane 10, Fig. S1A, L, Fig. S1B) by the wild type enzyme in the same time period, however with significant amounts of open circle form (OC, Fig. S1A, Fig. S1B) during the course of the reaction. The production of open circle DNA by the enzymes suggests that the cleavage rate is not significantly faster than the dissociation rate, therefore single cleavages tend to occur during the lifetime of the enzyme-DNA complex, rather than cleavage of both strands. This is especially true of the mutant enzyme. In addition, the cleavage of pMLE2 by P27W SgrAI is slower than that by the wild type enzyme, consistent with the measured 2-3 fold slower single turnover rate constant for DNA cleavage (Table 3). Single exponential functions were fit to the decrease in supercoiled DNA by wild type and P27W SgrAI enzymes giving rate constants of 0.077 and 0.019 min^-1^, respectively.

The cleavage of the plasmid containing two copies of the primary site sequence is, as reported, accelerated relative to that of the single site containing plasmid, when incubated with the wild type enzyme (Fig. S1B). Only a small amount of open circle (OC, Fig. S2A, S2B) and linear (L, Fig. S2A, S2B) DNA is seen, being converted to the final products of dual cleavage (P1, P2, Fig. S2A, S2B) after only 20 minutes of incubation. After only one minute, 74% of the supercoiled DNA is cleaved, compared to the negligible amount found after 1 minute in the case of the single primary site containing plasmid. Attempts to fit the decrease in supercoiled DNA to a single exponential function were unsuccessful; the decrease is at least bimodal with a fast decrease with a rate constant of 1.4 min^-1^, and a slower phase with a rate constant of 0.22 min^-1^. Both rate constants are greater than that obtained with the single site plasmid (0.077 min^-1^), and the fast rate constant represents an 18 fold increase in the rate of DNA cleavage due to the presence of a second primary site in the plasmid DNA. The cleavage to the two products (P1, P2, Fig. S2A, Fig. S2B) is nearly complete by 20 minutes of incubation, whereas the cleavage of the single primary site containing DNA is only 87% complete after 60 minutes. In addition, after 5 minutes of incubation twice as much of the doubly cleaved plasmid DNA (P1, P2, Fig. S2A, S2B) is formed relative to the linear form produced by only a single cleavage (L, Fig. S2A, S2B). The early appearance of the products of dual cleavage is consistent with the previously reported concerted cleavage of plasmid DNA with two primary sites[1,2].

The cleavage of the plasmid with two primary sites (pMLE3) to linear DNA by P27W SgrAI (L, lanes 11-18, Fig. S2A, Fig. S2C) shows relatively little acceleration relative to the cleavage of the single primary site containing plasmid pMLE2 (L, lanes 11-18, Fig. S1A, Fig. S1C). Cleavage of supercoiled DNA (SC) to nicked DNA (OC) is faster when two primary site sequences are present on the same plasmid (Fig. S2C vs. Fig. S1C), as a result of the availability of twice as many sites available for cleavage, as well as possibly some acceleration of the cleavage reaction. The fitted single turnover DNA cleavage rate constant for the decrease in supercoiled DNA is 0.062 min^-1^, three-fold faster than that for the single primary site containing DNA pMLE2. Only nicked open circle (OC, lanes 11-18 Fig. S2A, Fig. S2C), or plasmid fully cleaved at one primary site (L, lanes 11-18 Fig. S2A, Fig. S2C), are formed by P27W SgrAI, without any visible products from dual cleavage (P1 or P2, Fig. S2A). The absence of the products from dual cleavage, with only those from single cleavages at one or both strands, is consistent with the absence of concerted cleavage of the two primary sites, and hence the absence of the formation of an activated oligomer of the enzyme on the plasmid DNA.

**References**

1. Bilcock DT, Daniels LE, Bath AJ, Halford SE (1999) Reactions of type II restriction endonucleases with 8-base pair recognition sites. J Biol Chem 274: 36379-36386.

2. Wood KM, Daniels LE, Halford SE (2005) Long-range communications between DNA sites by the dimeric restriction endonuclease SgrAI. J Mol Biol 350: 240-253.
